# Looking back at 2025: Strengthening primary care evidence for Malaysia, regional partners, and the global health community

**DOI:** 10.51866/ed0009

**Published:** 2025-12-01

**Authors:** Salim Hani

**Affiliations:** 1 MBBChBAO, MFamMed, PhD, Department of Family Medicine, Faculty of Medicine and Health Sciences, Universiti Putra Malaysia (UPM), Jalan Universiti 1, Serdang, Selangor, Malaysia. Email: hanisyahida@upm.edu.my

As we near the end of 2025, Malaysian Family Physician (MFP) continues to strengthen its role as a regional platform for high-quality primary care research. This year marks a meaningful milestone: MFP is now indexed in the Web of Science - Emerging Sources Citation Index (ESCI). As a result, all articles published in MFP from 2023 onwards are retrievable through Web of Science. This achievement is a reflection of the collective dedication of our authors, reviewers, and editorial team, led by Professor Dr Lee Ping Yein. It positions the journal for greater international visibility in the coming years.

Building on this momentum, we are preparing for an important development in the first quarter of 2026: the migration to a robust journal management system. This upgrade will streamline the submission and review process, enhance transparency, and provide a better overall experience for our contributors. Strengthening our editorial infrastructure is essential as we continue to receive increasing numbers of submissions from Malaysia, the wider region and diverse international health settings.

This year’s citation patterns highlight several high impact papers in MFP. Based on the Web of Science, the most cited article among MFP articles published in 2023 to 2025 is Lee et al.’s, *Use of ChatGPT in medical research and scientific writing,* which has gained 24 citations and continues to attract significant attention as AI becomes more integrated into academic practice.^1^ Case-based clinical learning also featured strongly: Mahmud et al.’s report on delayed hypersensitivity to allopurinol recorded 8 citations, reflecting sustained clinical relevance.^2^

Meanwhile, original research addressing chronic disease and health systems also performed well. Chin et al.’s study on medication adherence among patients with type 2 diabetes received 7 citations,^3^ and Tabrizi et al.’s systematic review on accreditation of primary healthcare centres accumulated 6 citations,^4^ highlighting interest in quality improvement and health system performance. In addition, Banerjee et al.’s mixed-method study on adolescent reproductive and sexual health services (5 citations),^5^ and Ban et al.’s SABINA III asthma prescribing analysis (4 citations) contributed to shaping discussions on adolescent care and respiratory health.^6^

Together, these contributions reflect a clear trajectory: primary care research across Malaysia and the region is becoming more methodologically rigorous, more policy-relevant, and more attuned to the lived realities of patients and their families.

As we look ahead to 2026, MFP will continue to champion high-quality research, strengthen reporting standards, and encourage work that addresses digital transformation, equity, chronic disease management, and patient-centred care. With Web of Science - ESCI indexing in place and a new journal management system on the horizon, we are well positioned to support our research community and amplify their scholarly contributions on the global stage.

To our authors, valuable reviewers, and avid readers, I would like to thank you for your continued support. Your contributions are shaping MFP into a stronger and more visible voice for primary care in Malaysia and beyond. I look forward to another year of meaningful, practice-relevant research that strengthens primary care.
